# Efficacy of venous access placement at a pre-hospital scene in severe paediatric trauma patients: a retrospective cohort study

**DOI:** 10.1038/s41598-020-63564-w

**Published:** 2020-04-14

**Authors:** Tadashi Ishihara, Yutaka Kondo, Hiroshi Tanaka

**Affiliations:** Department of Emergency and Critical Care Medicine, Juntendo University, Urayasu Hospital, Urayasu, Chiba Japan

**Keywords:** Health care, Paediatrics, Paediatric research

## Abstract

Purpose: Aside from severe traumatic brain injury, uncontrolled bleeding and corresponding haemorrhage shock are the leading causes of traumatic deaths. No established recommendations exist about venous access placement for severely injured, bleeding children at a pre-hospital scene. This study sought to evaluate the association between pre-hospital venous access placement and mortality in a paediatric trauma population by analysing the Japan Trauma Data Bank (JTDB). Methods: This epidemiologic study compared the outcomes of severe traumatic paediatric patients with or without venous access placement at a pre-hospital scene. Data were obtained from JTDB from 2004 to 2015. Results: Of 4,109 patients who met our inclusion criteria, 144 patients received venous access placement and 3,965 patients did not. The probability of survival was lower in the venous access group than in the no access group (0.90 [0.67–0.97] vs. 0.97 [0.90–0.99], p < 0.01). After multivariable logistic analysis, venous access placement did not improve survival to hospital discharge (odds ratio = 1.40, confidence interval = 0.32–6.15, p = 0.653). Conclusions: The probability of survival was lower in the venous access group than in the no access group. Survival outcome at discharge was not affected by venous access placement at a pre-hospital scene.

## Introduction

Trauma is a major cause of death in children. Aside from severe traumatic brain injury, uncontrolled bleeding and corresponding haemorrhagic shock are the leading causes of traumatic deaths^1–4^. Haemorrhagic shock is responsible for 30–40% of trauma deaths, and of these deaths, 33–56% occur during the pre-hospital period^5,6^. Moreover, haemorrhagic shock is recognized as the leading cause of trauma death in the initial 24 hours after hospital admission^7^. Traditionally, management of haemorrhagic shock, including early rapid intravenous fluid resuscitation in the pre-hospital scene or during transport to a definitive care facility, has been considered important in both paediatric and adult patients. Because pre-hospital resuscitation with fluid replacement can be considered as a lifesaving intervention, severely injured patients are more likely to receive fluid resuscitation^8,9^.

The current guidelines of the German Trauma Society for the most severely injured patients and the latest guidelines of the European Resuscitation council do not mention volume replacement therapy in children at the pre-hospital scene^10^. No randomized trial has evaluated pre-hospital fluid resuscitation in the paediatric trauma population.

Venous access placement is needed for fluid resuscitation; however, there is another argument that venous access placement at a pre-hospital scene indirectly increases the risk of mortality in the injured patient by prolonging pre-hospital scene times and delaying patient transport to a definitive trauma care facility^11–13^.

However, all of the aforementioned studies involved adult patient cohorts. Concerning severely injured, bleeding children, there are currently no clear recommendations or studies with a high level of evidence^14^.

This study sought to evaluate the association between pre-hospital venous access placement and mortality in a paediatric trauma population by analysing of the Japan Trauma Data Bank (JTDB).

## Results

**Figure 1:**
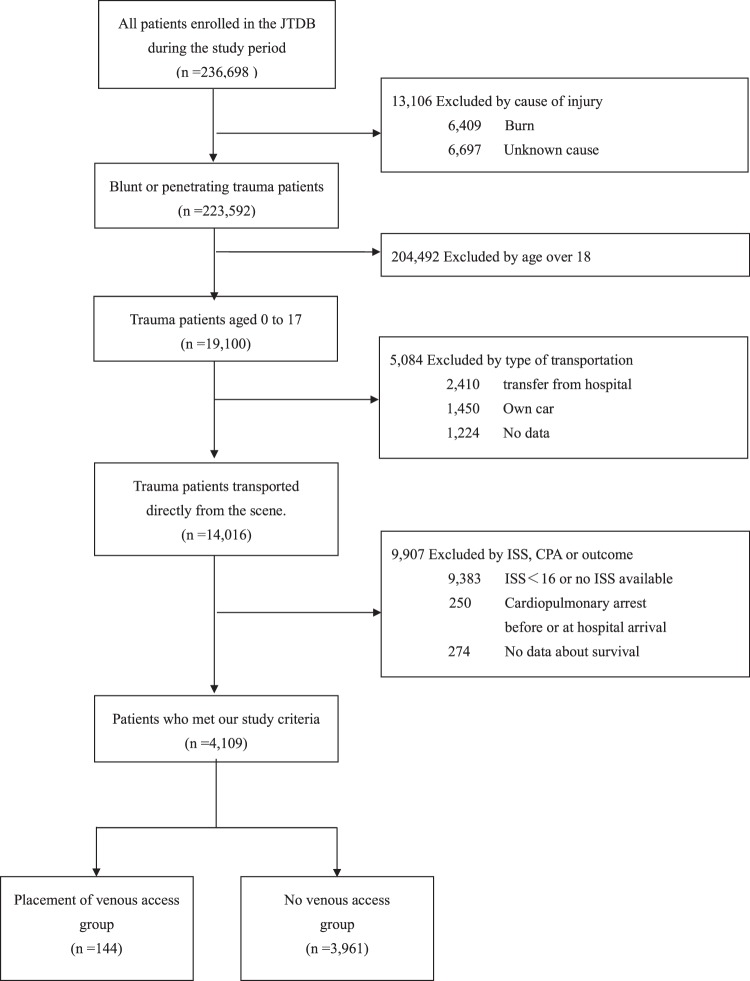
Inclusion criteria of the study.b JTDB: Japan Trauma Data Bank, ISS: Injury Severity Score, CPA: cardiopulmonary arrest.

Figure 1 shows the inclusion criteria of this study. Ultimately, 4,109 patients met our criteria. One hundred and forty-four patients received venous access placement, and 3,965 patients did not. Although there was no significant difference in age, sex, and blunt trauma between the two groups, the time from accident to hospital arrival was significantly longer in the venous access group than in the no access group (55.7 ± 19.6 versus [vs.] 43.5 ± 18.6, minutes). Traffic accident was the leading cause of injury followed by falls and sports. There was no significant difference in cause of injury and pre-hospital vital signs between the two groups (Table 1).

**Table 1:** Patient’s characteristics.

Characteristics	Venous access group (n = 144)	No access group (n = 3,965)	p-values
Age (years), mean ± SD	11.4 ± 4.8	11.3 ± 5.3	0.874
Gender (male, %)	103 (71.5)	2751 (69.3)	0.648
Type of trauma (blunt, %)	142 (98.6)	3925 (99.0)	0.981
Time from accident to hospital arrival	55.7 ± 19.6	43.5 ± 18.6	<0.01^*^
**Cause of injury**			
Traffic accident (%)	112 (77.7)	2770 (69.9)	0.052
Fall (%)	22 (15.3)	901 (22.7)	0.045
Sport (%)	5 (3.5)	101 (2.5)	0.674
Others (%)	5 (3.5)	193 (4.9)	0.569
**Pre-hospital vital signs**			
SBP (mmHg)	122 ± 26	121 ± 23	0.505
DBP (mmHg)	73 ± 20	71 ± 19	0.259
HR (beats/min)	100 ± 28	99 ± 27	0.616
RR (breaths/min)	26 ± 12	26 ± 9	0.896

^*^p < 0.01.

SD: standard deviation, SBP: systolic blood pressure, DBP: diastolic blood pressure,

HR: heart rate, RR: respiratory rate.

Table 2 shows injury characteristics. The Abbreviated Injury Scale (AIS) score for head injury was significantly higher in the venous access group than in the no access group. Patients in the venous access group had more severe trauma scores with a higher Injury Severity Score (ISS) (median [IQR]: 26 [20–38] vs. 22 [17–29], p < 0.01) and lower Revised Trauma Score (RTS) (6.08 [5.03–7.55] vs. 7.55 [5.97–7.55], p < 0.01) than those in the no access group. The probability of survival was also lower in the venous access group than in the no access group (0.90 [0.67–0.97] vs. 0.97 [0.90–0.99], p < 0.01).

**Table 2:** Injury characteristics.

	Venous access group (n = 144)	No access group (n = 3,965)	p-values
**AIS**			
Head (n = 3,236)	4.5 (4–5)	4 (4–5)	<0.01^*^
Face (n = 1,129)	2 (1–2)	1 (1–2)	<0.01^*^
Neck (n = 55)	3 (3–3)	1 (1–2.75)	0.238
Thorax (n = 1,795)	3 (3–4)	3 (3–4)	0.524
Abdomen (n = 1,795)	2.5 (2–3.25)	3 (2–3)	0.81
Spine (n = 484)	2 (2–3)	3 (2–4)	0.284
Upper extremity (n = 1,027)	2 (1–2)	2 (1–2)	0.697
Lower extremity (n = 1,388)	3 (2–3)	2 (2–3)	0.026
Others (n = 294)	1 (1–1)	1 (1–1)	0.46
ISS	30 ± 12.1	24.9 ± 10.3	<0.01^*^
RTS	5.98 ± 1.74	6.67 ± 1.67	<0.01^*^
TRISS	0.764 ± 0.274	0.874 ± 0.224	<0.01^*^

^*^p < 0.01.

AIS: Abbreviated Injury Scale, ISS: Injury Severity Score, RTS: Revised Trauma Score TRISS: Trauma and Injury Severity Score.

Table 3 shows the interventions performed at pre-hospital scenes and in the hospital. The administration of oxygen was the most popular intervention in both groups, followed by intubation. A significant higher proportion of patients in the venous access group compared to the no access group received oxygenation, ventilation, intubation, and a nasal airway tube at a pre-hospital scene (p < 0.01). A significantly higher number of patients in the venous access group than in the no access group received transfusion in the hospital within 24 hours after injury (51 [35.4%] vs. 735 [18.5%], p < 0.01).

**Table 3:** Intervention.

Intervention	Venous access group (n = 144)	No access group (n = 3,965)	p-values
**Pre-hospital**			
Received oxygen (%)	122 (84.7)	2,677 (67.5)	<0.01^*^
Bag mask ventilation (%)	24 (16.7)	58 (1.5)	<0.01^*^
Intubation (%)	34 (23.6)	156 (3.9)	<0.01^*^
Nasal airway tube (%)	4 (2.8)	8 (0.2)	<0.01^*^
**Hospital**			
Transfusion within 24 h (%)	51 (35.4)	735 (18.5)	<0.01^*^

^*^p < 0.01.

TAE: transcatheter arterial embolization.

Table 4 shows patients’ outcome. Two patients in the venous access group and 93 patients in the no access group died in the emergency room. Finally, as the main outcome, 25 (17.4%) patients in the venous access group and 375 (9.5%) patients in the no access group died, and this difference was statistically significant.

**Table 4:** Patient outcome.

	Venous access group (n = 144)	No access group (n = 3,965)	p-values
**Disposition at ER**			
Survived (%)	142 (98.6)	3872 (97.7)	0.64
Critical care center (%)	130 (90.2)	3219 (81.2)	<0.01^*^
General ward (%)	7 (4.9)	555 (14.0)	<0.01^*^
Transportation (%)	5 (3.5)	98 (2.5)	0.629
Died (%)	2 (1.4)	93 (2.3)	0.64
**Disposition at discharge**			
Survive (%)	119 (82.6)	3590 (90.5)	<0.01^*^
Home (%)	69 (47.9)	2582 (65.1)	<0.01^*^
Transportation (%)	50 (34.7)	1008 (25.4)	0.0159
Died (%)	25 (17.4)	375 (9.5)	<0.01^*^

^*^P < 0.01.

ER: emergency room

The results of multivariable logistic for survival outcome are shown in Table 5. Venous access placement did not improve survival to hospital discharge (odds ratio = 1.40, confidence interval = 0.32–6.15, p = 0.653).

**Table 5:** Logistic regression analysis.

	Survival OR	95% CI	p-value
Survival to hospital discharge (unmatched)	0.50	0.32 to 0.78	<0.01^*^
Survival to hospital discharge (matched)	1.40	0.32 to 6.15	0.653

OR: odds ratio, CI: confidenceinterval.

## Discussion

One remarkable finding of this study is that venous access placement at a pre-hospital scene did not improve the mortality of severe traumatic pediatric patients. After matching the severity of the two groups, there was no significant difference in venous access placement at the pre-hospital scene. Adversely, time from accident to hospital arrival was statistically longer in the venous access group than in the no access group. The power analysis of this study is over 0.8; thus, it has sufficient power to detect a meaningful difference.

Some studies reported that performance of pre-hospital procedures, including intubation or placement of intravenous access, was beneficial for the critically injured patients with blunt trauma in rural area even with prolonged transportation time^15^. Other studies support the performance of pre-hospital procedures, but these studies included patients with blunt and head injuries in rural areas^15–22^. Although these reports seems to justify the performance of prehospital procedures in patients in rural areas with prolonged transport times, the pre-hospital procedures have not been substantiated in an urban trauma population with accessible and rapid transportation to trauma centers^23–25^. In Japan, there is substantial EMS, included equiopped organized ambulance or medical helicopter with emergency physicians; so thus, the trauma patients can be easily transported to the critical care center.

In a previous study, Seamon reported that of all the measured clinical characteristics, the only independent risk factor found to adversely influence survival before hospital discharge was the performance of pre-hospital procedure^11^. By assessing pre-hospital procedures, including placement of intravenous access, the authors demonstrated that pre-hospital procedures provide no survival benefit to critically injured trauma patients. Furthers, patients were 2.63 times less likely to survive each procedure performed at the pre-hospital scene^11^.

Other prior studies reported that venous access placement at the pre-hospital scene increased pre-hospital time by 5 minutes, which was exacerbated by the longer transport time^26^. Smith reported that placement of intravenous access in a pre-hospital setting, takes 8.6–12.6 minutes, and McSwain reported that it took 11 minutes to place an intravenous line in a pre-hospital setting^23,27^. These reports suggest that venous access placement should not be a legitimate reason to delay patient transport to a definitive care facility. Further, delayed arrival to the hospital and the performance of any procedure before arrival to the trauma center were independent risk factors for death^11^. In addition, this previous report suggests that survival of critically injured trauma patients may be improved if pre-hospital intervention is minimal and procedures are restricted until arrival at a trauma center^11^. Although in our study, there was no difference in outcome, time from accident to hospital arrival was 8 minutes longer in the venous access group than in the no access group. Our data did not determine whether intravenous access should be performed at a pre-hospital setting or not but, it suggests that that the pre-hospital procedures should be minimal so as not to delay arrival at a critical care center for definitive intervention. Further randomized studies to determine the efficacy of venous access placement are needed.

There were several limitations to this study. First, we conducted a retrospective analysis; therefore, only associations among the given data could be described. Second, there were cases of missing data, and this might have generated information bias. Third, patients in our study might differ significantly in their injury epidemiology; the most commonly injured body part was the head, and this might be related to the physiological response to traumatic injury. Fourth, some other interventions at the pre-hospital scene may affect mortality. Fifth, although we adjusted for several measured cofounders in the multivariable regression analysis, unmeasured cofounders may have remained. Sixth, the number of patients in the venous access group was very small, because EMS personnel are not allowed to perform venous access placement in patients younger than 15 years of age. Finally, the transportation time could be affected by the distance from the accident area to the hospital. There might be differences in the medical environment provided by regional emergency medical service between urban and rural areas. Although, more than 90% of the hospitals that participate in the JTDB are tertiary emergency medical centers that have been certified by the Japanese Ministry of Health, Labor, and Welfare as being competent in providing trauma care, there may have been institutional bias.

In conclusion, this is the first report from Japan about the efficacy of venous access placement at the pre-hospital scene in paediatric trauma patients. Although the probability of survival was lower in the venous access group than in the no access group, the survival outcome at discharge was not affected by venous access placement at the pre-hospital scene. In the future, a randomized controlled trial on the efficacy of venous access placement is required.

## Methods

### Study design and data collection

This retrospective cohort study compared the outcomes of severe traumatic paediatric patients with or without venous access placement at the pre-hospital scene. Data were obtained from the JTDB, which was established in 2003 by the Japanese Association for the Surgery of Trauma (Trauma Registry Committee) and the Japanese Association for Acute Medicine (Committee for Clinical Care Evaluation) to assure the quality of trauma care in Japan. Between 2004 and 2015, 244 hospitals participated in the JTDB, 90% of which were tertiary emergency centers^28^. The JTDB is not open access data. Tertiary emergency centers in Japan are equivalent to level I or II trauma centers in the United States. The JTDB includes patient characteristics, injury type, cause of injury, transportation type, pre-hospital vital signs and treatment, AIS, ISS, disposition at emergency department (ED), disposition at discharge and information about the time of accident occurrence, pre-hospital care, and hospital arrival. The RTS and probability of survival based on the Trauma and Injury Severity Score (TRISS) were calculated using these data.

### Ethical approval and consent to participate

The ethics committee of Juntendo University Urayasu Hospital approved the JTDB data analysis (approval number: 29–061). The requirement for patient or parent consent was waived by the ethics committee of Juntendo University Urayasu Hospital, as this was an epidemiologic study that used anonymized data. We obeyed the STROBE statement to provide this study.

### Selection of patients

Overall, 236,698 patients were registered in the JTDB between April 2004 and March 2015. Paediatric patients younger than 18 years of age were included in this study. We identified 14,202 patients with blunt or penetrating trauma who were transported via emergency medical services (EMS, an ambulance without a physician), an ambulance with a physician or helicopter with a physician, directly from the injury site. EMS personnel are not allowed to perform venous access placement in patients younger than 15 years of age by Japanese emergency service system.

Of these, patients with an ISS ≥ 16 were selected for this study because of the requirement for specialized trauma care^29^. Patients with cardiopulmonary arrest before hospital arrival, those with burns, and those who were transported from another hospital or arrived at the hospital by themselves were excluded from this study. Cases with missing data regarding survival, pre-hospital vital signs (systolic blood pressure, diastolic blood pressure, respiratory rate, and heart rate), and pre-hospital procedure were also excluded.

### Outcome measures

The primary outcome of this study was survival to hospital discharge. The second outcomes were morality in the ED, time from accident to hospital arrival, rate of surgery, and rate of transfusion within 24 hours.

### Statistical analysis

The minimum required sample size was calculated from the mortality, then the power analysis was performed. To display the patient data, the mean ± standard deviation or median with interquartile range (IQR) were used for numerical variables. The t-test was used to compare the means of continuous variables between patients with venous access placement (venous access group) and those without venous access placement (no access group). The chi-square test was used to compare frequencies between the two groups. To assess the independent effect of venous access placement at the pre-hospital scene on study endpoints, multivariable logistic regression analysis of survival was performed. Covariates were carefully selected based on the assumption that they were not affected directly by the intervention. TRISS and the time from accident to arrival at the hospital were included as variables of multivariable logistic analysis. Differences were considered significant when the P-value was less than 0.01.
